# A Test of Reliability: Cranial Rhythmic Impulse for Distant Diagnoses

**DOI:** 10.7759/cureus.68219

**Published:** 2024-08-30

**Authors:** Luis A Alvarez, Andrew C Cook, Connor P Sweeney, Madison R Walter, Shannon C South, Nicole Myers, David Boesler, Steven Ma, Kevin Thomas, Thomas A Quinn

**Affiliations:** 1 Osteopathic Manipulative Medicine, Lake Erie College of Osteopathic Medicine, Bradenton, USA; 2 Osteopathic Manipulative Medicine, Lake Erie College of Osteopathic Medicine, Seton Hill, USA

**Keywords:** cranial rhythmic impulse (cri), somatic dysfunction, osteopathic manipulative medicine (omm), intra-rater reliability, inter-rater reliability, osteopathic cranial manipulative medicine, osteopathic manipulative techniques (omt), distant diagnosis, primary respiratory mechanism

## Abstract

Context

The osteopathic cranial field suggests that cranial rhythmic impulse (CRI) can be used to examine distal segments. However, there is a lack of research on the reliability of using CRI to diagnose other distal segments. This study aims to evaluate the effectiveness of using the cranial vault hold compared to traditional osteopathic diagnostic techniques to diagnose somatic dysfunctions at the following segments: atlantooccipital joint (OA), atlantoaxial joint (AA), cervical-4 (C4), cervical-7 (C7), thoracic-6 (T6), thoracic-12 (T12), lumbar-3 (L3), sacrum, left innominate, right fibular head, and left radial head.

Objective

To determine if palpation of CRI can reliably detect somatic dysfunctions in multiple distal segments.

Methods

The study compared osteopathic physicians' diagnoses of specific segments (OA, AA, C4, C7, T6, T12, L3, sacrum, left innominate, right fibular head, and left radial head) using the cranial vault hold and direct palpation. Two osteopathic neuromusculoskeletal medicine experts (cranial group) diagnosed distal segments via the cranial vault hold, while board-certified osteopathic physicians (confirmatory group) used direct palpation. We recruited 44 second-year osteopathic medical students and osteopathic physicians via a school-wide email. Each participant lay supine on a massage table for diagnosis. A neuromusculoskeletal expert, with a scribe, diagnosed the segments using the cranial vault hold. The process was repeated by a second neuromusculoskeletal expert with another scribe. Two osteopathic physicians then diagnosed the same subjects using direct palpatory techniques. Both osteopathic physicians had to agree on a diagnosis for the segment, or it was excluded from comparison. Cohen’s kappa coefficient measured inter-rater reliability between the cranial and confirmatory groups.

Results

Cranial physician 1 provided all 484 diagnoses, while cranial physician 2 provided 152. Cranial physician 1 showed positive agreement with the confirmatory group (κ>0) in 2/11 (18.2%) segments: T12 and left innominate (κ=0.009 and 0.007). Cranial physician 2 showed positive agreement (κ>0) in 4/11 (36.4%) segments: OA, AA, C4, and left innominate (κ=0.050, 0.031, 0.130, and 0.154). Inter-rater reliability between cranial physicians showed positive agreement in 6/11 (54.5%) segments: OA, AA, C4, sacrum, left innominate, and right fibular head (κ=0.125, 0.022, 0.048, 0.036, 0.154, and 0.0261).

Conclusion

The positive kappa values, all between 0 and 0.2, indicate the inter-rater reliability for diagnosis with the vault hold is above random chance but has none to slight reliability. The kappa coefficients comparing both cranial physicians indicate positive agreement in six segments, supporting palpation of the same phenomena in six out of 11 (54.5%) segments. However, none of the positive kappa values were statistically significant (p>0.05) and the effect sizes were small, likely due to shared bias among the evaluators. We conclude our experiment suggests palpation of the cranium may not reliably diagnose distal segments. However, our experiment may support a connection between CRI and distal segment somatic dysfunctions. Considering diagnoses of certain segments are above random chance, more research is needed to confirm whether there is a connection between palpation of the CRI and the diagnosis of a distal somatic dysfunction.

## Introduction

During the early 20th century, William G. Sutherland, DO, pioneered the field of cranial osteopathy. Cranial osteopathy is built upon the concept of the “primary respiratory mechanism,” which states that the brain possesses an inherent motility that induces rhythmic pulsation of cerebrospinal fluid (CSF), as well as palpable movement of the cranial bones, intracranial membranes, and sacrum [1]. This mobility of the brain is termed the cranial rhythmic impulse (CRI). Sutherland suggested that the primary respiratory mechanism can be altered by manipulating the skull via light palpation, and further proposed that altering this mechanism could help treat somatic dysfunctions [2]. Current clinical applications of CRI and cranial osteopathy include treating infantile colic, expediting concussion recovery, and benefiting patients suffering from various lymphatic, neurovascular, or psychiatric issues [3-5].

A practitioner typically gauges a patient’s CRI as follows: while the patient lies supine, the practitioner sits at the head of the table, holds the patient’s head with their palms, and uses their fingers to contact the cranial bones. The practitioner’s second digits lie over the greater wings of the sphenoid bone over the patient’s temples, while their third, fourth, and fifth digits lie over the patient’s pterion, mastoid process, and occiput, respectively. This position is known as the cranial vault hold. With sufficient training, a gentle expansion and contraction of the patient’s head can be felt between the practitioner’s hands [5-7]. A patient’s CRI is most often gauged in order to assess somatic dysfunctions of the cranial bones, which can then provide the practitioner with insight regarding underlying causes or potential cranial osteopathic treatments. However, some cranial specialists have explored the possibility of diagnosing somatic dysfunctions at distant structures, including the vertebral column, pelvic bones, and bones of the extremities, while palpating the skull. Although this is not a typical application of CRI, assessment of its potential is beneficial to solidifying the capabilities of CRI for diagnosis and treatment. 

This study aims to assess the effectiveness of the cranial vault hold in comparison to traditional osteopathic diagnostic techniques for diagnosing somatic dysfunctions at the following segments: atlantooccipital joint (OA), atlantoaxial joint (AA), cervical-4 (C4), cervical-7 (C7), thoracic-6 (T6), thoracic-12 (T12), lumbar-3 (L3), sacrum, left innominate, right fibular head, and left radial head. By pursuing this objective, we can investigate the potential of utilizing the cranial vault hold for diagnosing somatic dysfunctions beyond the cranium, while also evaluating its performance in relation to conventional local osteopathic diagnostic techniques.

## Materials and methods

Study approval and participant selection

The researchers sought approval for this study from the Institutional Review Board (IRB) of Lake Erie College of Osteopathic Medicine (LECOM) under the IRB number 30-078, and it was approved without exemption. Informed consent forms were sent to participants via email upon enrollment in the study. Participants had the option to review and sign the consent forms before the study or upon arrival. A co-investigator oversaw the collection of participants' consent forms and demographic information and addressed any queries regarding the study. Participants who chose to take part in the study were offered a $20 gift certificate for the LECOM cafeteria.

The pool of participants was limited to second-year medical students and faculty familiar with both craniosacral and traditional osteopathic diagnostic techniques. Familiarity with the techniques ensured that only brief explanations were required for each of the diagnostic techniques performed during the experiment. Participants were recruited via a mass email sent to the campus’s class of 2025 student cohort and the Osteopathic Principles and Practices (OPP) Department faculty. This email contained information about the study date, location, time commitment, and compensation, as well as the contact information of the principal investigator and directions for how to sign up. The study took place on 01/10/2023 and data collection lasted for one week. Participants were exclusively recruited from the medical school. All willing medical school participants were eligible with no exclusion criteria implemented. Participants were asked to anonymously sign up for a time slot on a Google Sheet and email the designated co-investigator their name and the time slot they chose. The co-investigator then sent the participants a confirmation email with the informed consent form. A maximum of four participants could sign up for each time slot, and each participant in each time slot was assigned a number and letter for data collection to maintain anonymity. For example, the second participant in the first time slot would be given the notation “1b.” Each participant’s age and sex were also recorded.

Data collection and diagnostic procedure

At the beginning of each time slot, participants were escorted to large examination rooms, each with two osteopathic examination tables to accommodate two participants. Each participant was instructed to lie supine on an osteopathic examination table and to avoid moving unless instructed to. The participants would soon have osteopathic physicians diagnose somatic dysfunctions at the following segments, either through a cranial vault hold or through direct palpation of the segments: OA, AA, C4, C7, T6, T12, L3, sacrum, left innominate, right fibular head, and left radial head. Qualifications of the osteopathic physicians performing these diagnoses include two osteopathic physicians board certified in neuromusculoskeletal medicine by the American Osteopathic Association (AOA), as well as two osteopathic physicians who have applied osteopathic manipulative medicine (OMT) in a medical setting and teach OMT to osteopathic medical students.

First, one osteopathic neuromusculoskeletal expert tasked with performing a cranial evaluation would enter the room accompanied by a scribe and diagnose multiple somatic dysfunctions on each participant’s body using the cranial vault hold technique. The neuromusculoskeletal expert would state the diagnoses out loud, and the scribe would subsequently record their response for the appropriate segment diagnosed. Once each participant in the room had been evaluated, the first neuromusculoskeletal expert and scribe would exit the room. Then, the second neuromusculoskeletal expert and a second scribe would enter the room to diagnose the same somatic dysfunctions using the cranial vault hold in the same manner. Once finished, the second neuromusculoskeletal expert and scribe would leave the room. Of note, diagnoses of distal somatic dysfunction by the neuromusculoskeletal experts were recorded using specific osteopathic diagnostic terminology, such as "anterior left innominate" or "T12 extended, rotated right, and sidebent right." The neuromusculoskeletal experts did not describe restricted motion in a specific segment to the scribes in any other way.

Once each neuromusculoskeletal expert had diagnosed both participants and exited the room, two additional osteopathic physicians would enter the room with two scribes. These physicians would diagnose the same somatic dysfunctions using a regional approach and direct palpation. These physicians discussed their findings for each participant in order to reach a consensus for each diagnosis, as these diagnoses would be compared to the neuromusculoskeletal experts' diagnoses. If these two physicians could not agree on a specific diagnosis, the data point was discarded. If the examiners disagreed on the severity of a somatic dysfunction but agreed on the end diagnosis, the data point was kept.

A paper grading sheet was used for data collection. Each participant had one grading sheet that stayed with them throughout the experiment; all diagnoses for each physician were recorded on the same sheet to facilitate diagnosis comparison. The recorded diagnoses included the participant’s OA, AA, C4, C7, T6, T12, L3, sacrum, left innominate, right fibular head, and left radial head. An additional area of the grading sheet was used to indicate diagnoses that the confirmatory diagnostic physicians disagreed with and to indicate any data points that needed to be excluded.

Cohen’s kappa coefficient was used to measure diagnostic inter-rater reliability between four groups: cranial physician 1 compared to the confirmatory physicians, cranial physician 2 compared to the confirmatory physicians, the combined diagnoses of cranial physicians 1 and 2 compared to the confirmatory physicians, and cranial physician 1 compared to cranial physician 2. The following formula was used to calculate Cohen’s kappa: 𝜿=(P_o_- P_e_)/(1-P_e_), where P_o_ is the proportion of observed agreement between the cranial and confirmatory physicians, and P_e_ is the probability that a consensus on the diagnosis can be reached by random chance. Cohen’s kappa ranges from a value of -1 to +1, with negative values indicating the raters more perfectly disagreed, a value of zero indicating the raters agreed exactly due to random chance, and positive values indicating the raters had agreement above random chance. For the positive values, the following interpretation can be used to assess the strength of the inter-rater reliability: 0-0.20 (none-slight), 0.21-0.39 (minimal), 0.40-0.59 (weak), 0.60-0.79 (moderate), 0.80-0.90 (strong), 0.90+ (almost perfect) [8]. In addition, statistical significance was determined by calculating two-tailed p-values from z-statistics with 95% confidence intervals. A Bonferroni adjustment was done to the data to calculate an alpha level of 0.05/11=0.0045.

## Results

Forty-four participants were diagnosed by the two cranial physicians and the two confirmatory physicians at the following segments: OA, AA, C4, C7, T6, T12, L3, sacrum, left innominate, right fibular head, and left radial head. However, not all of the segments of each participant were included due to either a cranial physician being unable to diagnose a particular landmark or the confirmatory physicians not being able to agree upon a diagnosis (Table 1). Of the 44 participants, 24 were female (60%) and 20 (40%) were male. The average age of the participants was 27; the oldest participant was 82 while the youngest was 22. Cranial physician 1 was able to provide a diagnosis for 100% (n=484) of the segments. Cranial physician 2 was able to provide fewer diagnoses (n=152), (31.4%), ranging from 4.5% to 97.7% (n=2-43) of the time (Table 1). The confirmatory physicians largely agreed on each diagnosis, the least agreed upon being T6 (Table 1).

**Table 1:** Diagnostic rates of cranial and confirmatory physicians for specific anatomical landmarks in participants The data has been represented as a %. OA: atlantooccipital joint; AA: atlantoaxial joint; C4: cervical-4; C7: cervical-7; T6: thoracic-6; T12: thoracic-12; L3: lumbar-3

Landmark	Cranial 1 pre-exclusion	Cranial 1 post-exclusion	Cranial 2 pre-exclusion	Cranial 2 post-exclusion	Confirmatory group diagnoses in agreement
OA	44/44 (100%)	38/44 (86.4%)	12/44 (27.3%)	12/44 (27.3%)	38/44 (86.4%)
AA	44/44 (100%)	44/44 (100%)	29/44 (65.9%)	28/44 (63.6%)	43/44 (97.7%)
C4	44/44 (100%)	44/44 (100%)	5/44 (11.4%)	5/44 (11.4%)	43/44 (97.7%)
C7	44/44 (100%)	44/44 (100%)	4/44 (9.1%)	4/44 (9.1%)	43/44 (97.7%)
T6	44/44 (100%)	30/44 (68.1%)	3/44 (6.8%)	2/44 (4.5%)	19/44 (43.2%)
T12	44/44 (100%)	43/44 (97.7%)	2/44 (4.5%)	2/44 (4.5%)	42/44 (95.5%)
L3	44/44 (100%)	37/44 (84.1%)	2/44 (4.5%)	2/44 (4.5%)	36/44 (81.8%)
Sacrum	44/44 (100%)	42/44 (95.5%)	43/44 (97.7%)	41/44 (93.2%)	41/44 (93.2%)
Left innominate	44/44 (100%)	41/44 (93.2%)	35/44 (79.5%)	35/44 (79.5%)	41/44 (93.2%)
Right fibular head	44/44 (100%)	44/44 (100%)	14/44 (31.8%)	13/44 (29.5%)	43/44 (97.7%)
Left radial head	44/44 (100%)	44/44 (100%)	3/44 (6.8%)	3/44 (6.8%)	44/44 (100%)

After excluding the disagreements from the confirmatory group, the following number of diagnoses were used from cranial physician 1 in the calculation of the Cohen's kappas and p-values: OA (n=38, 86.4%), AA (n=44, 100%), C4 (n=44, 100%), C7 (n=44, 100%), T6 (n=30, 68.1%) T12 (n=43, 97.7%), L3 (n=37, 84.1%), sacrum (n=42, 95.5%), left innominate (n=41, 93.2%), right fibular head (n=44, 100%), and left radial head (n=44, 100%) (Table 1). This resulted in a total of 451 diagnoses from cranial physician 1 that we compared with Cohen’s kappa (“Cranial 1 Vs. Confirmatory” in Table 2). Cranial physician 1 agreed with the confirmatory group at variable percentages depending on the landmark, ranging from 0% to 45.5% (n=0-20) of the time (Table 2). Cranial physician 2 provided the following total diagnoses per segment, post-exclusion: OA (n=12, 27.3%), AA (n=28, 63.6%), C4 (n=5, 11.4%), C7 (n=4, 9.1%), T6 (n=2, 4.5%), T12 (n=2, 4.5%), L3 (n=2, 4.5%), sacrum (n=41, 93.2%), left innominate (n=35, 79.5%), right fibular head (n=13, 29.5%), and left radial head (n=3, 6.8%) (Table 1). This resulted in a total of 147 diagnoses from cranial physician 2 that we compared with Cohen’s kappa (“Cranial 2 Vs. Confirmatory" in Table 2). Cranial physician 2 also agreed with the confirmatory group at variable percentages, ranging from 0% to 62.9% (Table 2). Combined, the cranial physicians provided the following total diagnoses per segment: OA (n=50), AA (n=72), C4 (n=49), C7 (n=48), T6 (n=32), T12 (n=45), L3 (n=39), sacrum (n=83), left innominate (n=76), right fibular head (n=57), and left radial head (n=48) (Table 2). This resulted in a total of 599 diagnoses that we compared with Cohen’s kappa (“Combined” in Table 2). The cranial physicians agreed with the confirmatory group at the following range of percentages: 3.5% to 36.1% (Table 2). Upon comparing only similar diagnoses between each other, cranial physicians 1 and 2 provided the following total diagnoses per segment: OA (n=14), AA (n=29), C4 (n=5), C7 (n=4), T6 (n=3), T12 (n=2), L3 (n=2), sacrum (n=43), left innominate (n=37), right fibular head (n=14), and left radial head (n=3) (Table 2). This resulted in a total of 156 diagnoses that we compared with Cohen’s kappa (“Cranial 1 Vs Cranial 2” in Table 2). They agreed with the confirmatory group at the following range of percentages: 0% to 42.9% (Table 2).

**Table 2:** Inter-rater reliability among cranial physicians and confirmatory group for anatomical landmark diagnoses Italics indicate 𝜿>0. The diagnoses agreement is represented as a %. Cohen's Kappa has been represented as N. OA: atlantooccipital joint; AA: atlantoaxial joint; C4: cervical-4; C7: cervical-7; T6: thoracic-6; T12: thoracic-12; L3: lumbar-3; CI: confidence interval

Landmark	Cranial 1 Vs. Confirmatory Agreement (%)	Cranial 1 Vs. Confirmatory Cohen's Kappa	Cranial 1 Vs. Confirmatory Cohen's Kappa 95% CI	Cranial 1 vs Confirmatory p-value	Cranial 2 Vs. Confirmatory Agreement (%)	Cranial 2 Vs. Confirmatory Cohen's Kappa	Cranial 2 Vs. Confirmatory Cohen's Kappa 95% CI	Cranial 2 Vs. Confirmatory p-value	Combined Agreement %	Combined Cohen's Kappa	Combined Cohen's Kappa 95% CI	Combined p-value	Cranial 1 vs Cranial 2 Diagnosis Agreement (%)	Cranial 1 Vs. Cranial 2 Cohen's Kappa	Cranial 1 Vs. Cranial 2 Cohen's Kappa 95% CI	Cranial 1 Vs. Cranial 2 p-value
OA	6/38 (15.8%)	-0.012	-0.106-0.082	0.8006	2/38 (5.3%)	0.050	-0.112-0.212	0.5420	4/50 (8%)	-0.015	-0.097-0.068	0.7318	2/14 (14.2%)	0.125	-0.062-0.312	0.1902
AA	20/44 (45.5%)	-0.071	-0.360-0.218	0.6302	6/28 (21.4%)	0.031	-0.156-0.219	0.7416	26/72 (36.1%)	-0.048	-0.229-0.135	0.6140	6/29 (20.7%)	0.022	-0.160-0.204	0.812
C4	2/44 (4.5%)	-0.030	-0.097-0.036	0.3748	1/5 (20%)	0.130	-0.251-0.512	0.5030	3/49 (6.1%)	-0.017	-0.089-0.056	0.6530	1/5 (20%)	0.048	-0.370-0.465	0.8226
C7	7/44 (15.9%)	-0.061	-0.060-0.181	0.3250	0/4 (0%)	0	N/A	N/A	7/48 (14.6%)	-0.143	-0.060-0.160	0.3900	0/4 (0%)	-0.143	-0.143--0.143	N/A
T6	4/30 (13.3%)	-0.054	-0.202-0.094	0.4740	0/2 (0%)	0	N/A	N/A	4/32 (12.5%)	-0.054	-0.192-0.084	0.4420	0/3 (0%)	0	0-0	N/A
T12	5/43 (11.6%)	0.009	-0.098-0.117	0.8680	0/2 (0%)	0	N/A	N/A	5/45 (11.1%)	0.009	-0.09-0.111	0.8580	0/2 (0%)	0	0-0	N/A
L3	3/37 (8.1%)	-0.072	-0.174-0.031	0.1715	0/2 (0%)	0	N/A	N/A	3/39 (7.7%)	-0.069	-0.165-0.028	0.1660	0/2 (0%)	0	0-0	N/A
Sacrum	14/42 (33.3%)	-0.023	-0.241-0.196	0.8390	3/41 (7.3%)	0	-0.085-0.087	0.9883	17/83 (20.5%)	-0.008	-0.119-0.102	0.8800	6/43 (14%)	0.036	-0.080-0.152	0.539
Left innominate	5/41 (12.2%)	0.007	-0.107-0.120	0.9070	22/35 (62.9%)	0.154	-0.210-0.519	0.4070	27/76 (35.5%)	0.045	-0.115-0.204	0.5830	12/37 (32.4%)	0.154	-0.034-0.343	0.109
Right fibular head	0/44 (0%)	-0.970	-0.970--0.970	N/A	2/13 (15.4%)	-0.092	-0.345-0.161	0.4800	2/57 (3.5%)	-0.058	-0.11--0.005	0.0310	6/14 (42.9%)	0.026	-0.416-0.468	0.908
Left radial head	5/45 (11.1%)	-0.076	-0.186-0.036	0.1840	1/3 (33.3%)	-0.2	-1.160-0.760	0.6830	6/48 (12.5%)	-0.087	-0.203-0.029	0.1430	0/3 (0%)	-0.125	-0.125--0.125	N/A

Table 2 presents the inter-rater reliability between the cranial and confirmatory physicians and the kappa coefficient for each segment. Cranial physician 1 agreed by more than random chance with the confirmatory group ("Cranial 1 Vs. Confirmatory") on 2/11 of the segments, specifically T12 and left innominate (𝜿=0.009 and 0.007), indicating slight agreement (Table 2). On the other hand, cranial physician 2 agreed by more than random chance with the confirmatory group ("Cranial 2 Vs. Confirmatory") on 4/11 of the segments, specifically OA, AA, C4, and left innominate (𝜿=0.050, 0.031, 0.130, and 0.154) (Table 2). When both cranial physicians' data were combined ("Combined"), they agreed on 2/11 segments, specifically T12 and left innominate (Table 2).

Table 2 also illustrates the inter-rater reliability between the two cranial physicians ("Cranial 1 Vs. Cranial 2"). They agreed by more than random chance on 6/11 of the segments (𝜿>0) including OA, AA, C4, sacrum, left innominate, and right fibular head (𝜿=0.125, 0.022, 0.048, 0.036, 0.154, and 0.0261), indicating slight agreement. However, they did not agree on 5/11 (45.5%) of segments (C7, T6, T12, L3, and left radial head).

The only p-value <0.05 observed was the combined cranial 1 and 2 versus the confirmatory group at the right fibular head landmark with a p-value of 0.031 (Table 2). However, after a Bonferroni adjustment, with an alpha of 0.0045, this is no longer significant. There was no observed agreement as indicated by the negative Cohen's kappa. Additionally, the aforementioned positive Cohen's kappas in inter-rater and intra-rater reliability all contained zero in their 95% confidence intervals, suggesting a lack of agreement or statistical significance (Table 2).

Table 3 summarizes our results by showcasing the frequency of positive agreement, depending on which groups were compared to each other. Cranial physician 1, when compared to the confirmatory group, agreed by more than random chance (𝜿>0) in 2/11 segments. Cranial physician 2, when compared to the confirmatory group, agreed by more than random chance (𝜿>0) in 4/11 segments. When both cranial physicians' data were combined, they had a positive agreement in the 2/11 segments when compared to the confirmatory group. When the cranial physicians were compared to each other, they had a positive agreement in the 6/11 segments.

**Table 3:** Frequency of positive inter-rater reliability (κ>0) among the diagnosticians The data has been represented as a %. κ: Cohen's kappa

Diagnostician	Frequency κ>0
Cranial 1	2/11 (18.2%)
Cranial 2	4/11 (36.4%)
Combined	2/11 (18.2%)
Cranial 1 vs. cranial 2	6/11 (54.5%)

## Discussion

The goal of our experiment was to explore two questions: Can somatic dysfunctions distal to the cranium be diagnosed using the cranial vault hold, and if they can, how does it compare to traditional local osteopathic diagnostic techniques? We used Cohen’s kappa to evaluate whether the agreement between two raters is above random chance. A kappa coefficient greater than 0 indicates that the agreement between the raters is above chance, while a kappa coefficient of 0 indicates that the agreement is no better than chance. Additionally, the strength of the agreement can be assessed by its value. Values closer to +1 indicate greater agreement, whereas values closer to -1 indicate perfect disagreement. In addition, statistical significance was determined with a p-value less than 0.0045. The following segments were analyzed OA), AA, C4, C7, T6, T12, L3, sacrum, left innominate, right fibular head, and left radial head.

The cranial physicians agreed above random chance with the confirmatory group in 2/11 (18.2%) segments (T12 and left innominate). However, the strength of agreement among these segments was poor. While the positive Cohen's kappa values indicate agreement between the various groups, they were not statistically significant. Additionally, the 95% confidence intervals for these groups all contained zero. This shows a lack of evidence to show that the diagnoses of the tested distant segments could be accurately assessed via cranial vault hold.

With that in mind, cranial physicians 1 and 2 showed positive agreement in 6/11 (54.5%) segments (OA, AA, C4, sacrum, left innominate, and right fibular head) when compared to each other. This finding may support the idea that they were palpating the same phenomenon and applied it similarly to the six segments. However, the p-values were less than 0.0045 and the 95% confidence intervals contained 0, indicating they were not statistically significant. This lack of statistical significance and small effect size could be due to shared bias among the evaluators. The negative kappa coefficients for C7, T6, T12, L3, and the left radial head indicate that the cranial physicians were not consistent in these segments when compared to each other. It is important to note that there was an inability to compare agreement between T6, T12, and L3 due to inconsistencies in the number of diagnoses completed by cranial physician 2.

The cranial physicians did not perform the same number of diagnoses. It is thus more reasonable to analyze each cranial physician separately considering our large pool of collected diagnoses. Cranial physician 1 diagnosed 484 segments. Cranial physician 1 showed positive agreement in 2/11 (18.2%) segments (T12 and left innominate) when compared to the confirmatory group. Cranial physician 2 diagnosed 152 segments. Cranial physician 2 showed positive agreement for 4/11 (36.4%) segments (OA, AA, C4, and left innominate) when compared to the confirmatory group. Of these segments, cranial physician 2 diagnosed more than half the diagnoses for AA 29/44 (65.9%) and Ieft innominate 35/44 (79.5%) while only diagnosing 12/44 (27.3%) for OA and 4/44 (9.1%) for C4. Notably, cranial physicians 1 and 2 both had positive kappa coefficients for the left innominate when compared to the confirmatory group.

Cranial physician 2 had the strongest Cohen’s kappa overall, which was for diagnosis of the left innominate (𝜿=0.154) (Table 2). The strongest Cohen’s kappa of cranial physician 1 was for the diagnosis of T12 (𝜿=0.009) (Table 2). The inter-rater reliability between the cranial physicians was stronger than for their diagnoses versus the confirmatory group, indicating greater precision than accuracy. This finding may suggest that they observed the same phenomena. They agreed by above random chance at 6/11 segments (54.5%), with the strongest agreement at the left innominate (𝜿=0.154) (Table 3).

One unusual finding was the right fibular head kappa coefficient for cranial physician 1. The kappa coefficient suggests almost perfect disagreement (-0.970) between cranial physician 1 and the confirmatory group. One explanation for this strong disagreement could simply be a contradiction in what to look for during the vault hold for a right fibular head diagnosis. We believe this may offer insight into the validity of the palpation of CRI and its application to distal diagnosis. If a simple misunderstanding during the technique could almost consistently produce an unreliable result compared to the other diagnoses, then perhaps this supports at least the presence of the CRI phenomenon being translated to distal somatic dysfunctions.

There is a clear lack of research revolving around using the cranial vault hold to diagnose somatic dysfunctions distal to the cranium. As a result, we recognize that this may not be the intended application of CRI. A few past studies have examined the inter- and intra-rater reliability of palpating the CRI at the cranium and sacrum. Moran and Gibbons assessed simultaneous palpation at the cranium and sacrum of CRI. They found fair to good intra-rater reliability, but poor to nonexistent inter-rater reliability [9]. Sommerfield et al. examined the simultaneous assessment of the primary respiratory mechanism (PRM) at the head and sacrum. They did not find agreement beyond random chance for both intra- and inter-rater reliability [10]. Rogers et al. tested the simultaneous assessment of CRI at the head and feet. They found poor intra- and inter-rater reliability among the two groups [11].

Our results may not directly translate into immediate clinical applications. However, if future studies expand upon our findings and establish the validity of diagnosing distal somatic dysfunctions via CRI, certain clinical benefits may arise from palpation of the cranium rather than the direct segments. For example, consider patients who are immobile due to pain, sedation, or physical impairment. Diagnosing somatic dysfunctions via direct palpation would necessitate positioning these patients multiple times, which may be infeasible or painful for them. Utilizing palpation of the cranium to diagnose somatic dysfunctions could not only reduce the time needed for diagnosis but could be more practical and comfortable for immobile patients. Similarly, this method of diagnosis may prove beneficial in patients who speak a different language. For instance, asking a patient to lie still is much simpler than coordinating them into thoracic flexion or lumbar side bending. Overall, our research questions lay a foundation for exploring the capabilities of CRI, a field with clear gaps in research.

Future directions of this study could include applying our test of reliability to segments along fascial planes rather than junctional segments throughout the body. Continuing to examine the associations between CRI and the accurate diagnosis of distal somatic dysfunctions would lead to a deeper understanding of the impact of CRI on the body. This understanding can enable experts in osteopathic neuromusculoskeletal medicine to further develop and refine CRI as a diagnostic tool beyond its current primary application to the cranium.

Limitations

Our study compares the reliability of using the vault hold to diagnose distal segments when compared to traditional osteopathic diagnostic techniques. One limitation of our study may lie in having the two confirmatory physicians agree to a diagnosis rather than reporting diagnoses from each confirmatory physician even if they were distinct. Somatic dysfunctions can have a degree of subjectivity, which leads to disagreement in the first place. This may also be true for diagnosis via CRI. Of note, our study only used healthy subjects. Although healthy subjects typically have some degree of somatic dysfunction, it is not to the degree of clinical significance. Perhaps if unhealthy subjects were used, the degree of somatic dysfunction would be less subjective and the data between diagnosticians more consistent.

It is also important to consider the homogenous participant population as a limitation of this study. Only second-year medical students and faculty familiar with osteopathic diagnostic techniques were included as subjects. This restriction was established in order to avoid explaining specific diagnostic positioning to each subject and provide more time for diagnosis, this undoubtedly limits how well the results can be generalized. Ideally, this study could be performed with members of the public or actual patients.

During the experiment, one neuromusculoskeletal expert took significantly longer to diagnose than the other. This inconsistency was due to cranial group physician 2 not diagnosing a landmark if they did not feel confident in their diagnosis. In contrast, cranial group physician 1 diagnosed all segments but reported significant mental fatigue by the end of the experiment. The reduction in sample size for cranial group physician 2 resulted in an inability to assess their Cohen’s kappas for C7, T6, T12, and L3. A larger sample size plus utilizing more neuromusculoskeletal experts would likely allow sufficient data points to compare all segments in future studies.

There were clear differences in the performance between the two neuromusculoskeletal experts. We recognize that the use of the cranial vault to detect CRI is a technique that requires a high degree of palpatory skill. A skill gap may be responsible for the inconsistency in results between the two cranial group physicians. To avoid this discrepancy, replication of this project should be done with more neuromusculoskeletal experts, as mentioned above.

## Conclusions

The positive kappa coefficient values obtained ranged from 0 to 0.154, indicating that the inter-rater reliability for diagnosing these segments using the vault hold is slightly above chance, but still demonstrates only none to slight agreement. None of these were statistically significant after the Bonferroni adjustment. When comparing both cranial physicians, the kappa coefficient indicates positive agreement in six out of 11 segments (54.5%), supporting the palpation of the same phenomena in those specific cases. Our experiment thus leads us to the conclusion that palpation of the cranium may not reliably diagnose distal somatic dysfunctions, however, a potential association between CRI and distal segments may still exist. However, these finding's effect sizes were small and not statistically significant, possibly due to shared information bias. Although some landmark diagnoses showed results in an above-chance agreement, further research is necessary to confirm the relationship between palpating the CRI and diagnosing distal somatic dysfunctions. The clinical utility of reliably diagnosing distal somatic dysfunctions using CRI may include cutting down on diagnosis time, reducing the impact of language barriers on diagnosis, and providing a more feasible and comfortable method of diagnosis for physically impaired patients. Our experiment lays a foundation for investigating the capabilities of CRI in future studies. This exploration has the potential to broaden the application of CRI and enhance its effectiveness in diagnosing various somatic dysfunctions throughout the body, emphasizing a core tenet of osteopathy: the body is a unit.
